# Talking about the Birth Family since the Beginning: The Communicative Openness in the New Adoptive Family

**DOI:** 10.3390/ijerph19031203

**Published:** 2022-01-21

**Authors:** Alessandra Santona, Giacomo Tognasso, Carla Luisa Miscioscia, Daniela Russo, Laura Gorla

**Affiliations:** 1 Department of Psychology, University of Milano-Bicocca, 20126 Milan, Italy; alessandra.santona@unimib.it (A.S.); l.gorla8@campus.unimib.it (L.G.); 2 CIAI—Centro Italiano Aiuti all’Infanzia, 20142 Milan, Italy; carla.miscioscia@ciai.it (C.L.M.); daniela.russo@ciai.it (D.R.)

**Keywords:** adoption, communicative openness, birth family, adoptive families

## Abstract

Communicative openness within the adoptive family changes over time and helps the child explore his/her history. We aimed to evaluate whether adoptive families communicate about specific adoption-related themes from the beginning of their lives as a family. We created an instrument to track the communication process during the first year of adoption, involving a sample of 537 internationally adopted children (313 males, 224 females, mean age of adoption: 4.9 years) at two time points: six (T1) and twelve (T2) months after adoption. Our results suggest that in the first year of placement, children express memories about the past but tend to not speak about their birth families. We discovered a significant difference (Wald test = 4.889; *p* = 0.027) in communication about the biological family between the two points. The presence of adoptive parents who speak about it impacts the child’s questions about the past (exp (B) = 2.452, *p* = 0.006) and whether the child speaks about his/her biological family (exp (B) = 2.373; *p* = 0.017). Then, in the first year of adoption, the presence of an adoptive parent who communicates openly helps the child to ask questions and share his/her thoughts.

## 1. Introduction

Communicating about adoption has always been considered an essential aspect for adoptive families [1,2,3,4].

Researchers have defined communication about adoption-related themes as openness in adoption [2]. Communicative openness (CO) involves looking at all adoption issues [1,5,6,7], and changes over time; it is a dynamic and flexible process. For this reason, it is necessary to study its development and how it evolves throughout the adoptive family’s life cycle [8,9].

CO is not only an exchange of information between the adoptive parents and the adopted child, but also consists of feelings, deep meanings, and emotions strictly tied to the adoption itself [10]. Many aspects are discussed together in the CO process: the child’s past life with and without the birth family, life in institutions, the adoptive parents’ desire to meet the child, and the first period together as a new family [11].

Several studies have drawn attention to CO’s positive effects on adoptees. Indeed, open communication is associated with better psychological adjustment, fewer behavioral problems, and higher self-esteem. Moreover, it helps adopted children create a more positive adoption identity and promotes adequate emotion regulation [12,13,14,15].

For all these reasons, studying the process of communication among adoptive family members is crucial, and there are a growing number of studies focused on this theme. However, several aspects remain unexplored [10]. To the best of our knowledge, there is a paucity of studies that analyze when adoptive families start to communicate about adoption-related issues. Choosing when to begin to communicate may be difficult for adoptive parents, so they often seek support through post-adoption services [16]. Indeed, for adoptive parents, talking about the child’s past life means sharing information about the life-changing and traumatic events that the child has experienced (i.e., abandonment, loss of their birth family, departure from their birth country in the case of international adoption). Moreover, it requires parents to support their child in giving meaning to these events and to guide the child in an emotionally challenging process of integration between past and present [17,18,19,20]. 

The emotional connotation of CO could especially intimidate adoptive parents in the first period of adoption when the adoptive family is engaged in building attachment bonds between its members [17]. For this reason, adoptive parents often wonder if it is possible to talk about the child’s past and birth family from the beginning. Nevertheless, adoption involves the loss of the birth family and entry into a new family, so there is a strong connection between the birth family and the adoptive family [11,18] from the beginning.

Given all these aspects, it is crucial to better understand the development and timing process of open communication.

Since the adoptive family is always strictly connected with the biological family, even when unknown and not physically present in the child’s life [21,22], the birth family and the child’s separation from that family need to be addressed through open communication. Indeed, adopted children often think about the decision their birth parents made to give them up for adoption, ask themselves whether this decision was made intentionally or unavoidably, try to imagine their birth parents’ motives and feelings [5,11,23] and, often when going through adolescence, start to seek information about their birth parents. For this reason, several studies have scrutinized the contact between adolescent or adult adoptees and their birth families [3,20,24,25,26,27,28], with few studies focused on how the birth family is discussed within the adoptive family when the adoptee is still a child.

The child’s age changes how he/she comprehends adoption-related themes and integrates his/her past and present experiences [5]. In fact, children aged between 3 and 6 are not able to deeply understand what being adopted means but, if they are in a family where there is open communication about adoption, they learn the language of adoption, and memorize and acquire the terms related to adoption. Real awareness of the meaning of adoption starts between 6 and 12 years due to the development of new cognitive competencies, more curiosity about one’s origins, and new questions about one’s birth family [5,11].

Both having contact with one’s birth family and being able to gain more information or communicate about it have positive effects on the adoptee’s development if he/she is part of a caring, nurturing adoptive family that can encourage, support, or begin a dialog regarding the birth family. The role of adoptive parents is then negotiating and mediating the adopted child’s contact with the birth family if there is contact between the birth family and the adoptee, or supporting the adoptee in giving meaning to his/her loss and separation from the birth family [29]. Adopted children or adolescents who perceive their adoptive parents as being supportive and emotionally close to them [30] tend to be satisfied with the contact they have with their birth family, or understand more aspects about their adoption and their relationships with their birth family [31]. 

These positive effects are present even though there is no direct contact between the adopted child and their birth relatives. Sharing information and thoughts about their birth family with their adoptive family allows the child to express his/her feelings and to integrate his/her past with his/her present [13].

Adoptive parents need to guide the child in his/her discovery of his/her past and roots, so they are asked to speak both about the child’s past and biological family to help the child fully understand his/her history [4]. In adoptive families where there is no dialog regarding the child’s past life, the adoptee tends to suffer more psychological problems and struggles to create an integrated self-identity [18,29].

### 1.1. Italian Context 

Italy is the second country in the world, after the United States, with the highest number of adopted children. In 2019, 1032 children were internationally adopted in Italy, with 2.644 new requests for international adoption being sent to the Juvenile Court [32]. Comparing intranational and international adoption, the latter represents 58% of the total. The choice to present data from 2019 is tied to the effects of COVID-19, because the pandemic situation slowed down international adoption procedures. 

Internationally adopted children who arrived in Italy in 2019 were 6.8 years old on average and 59% (*N* = 395) were classified as having “special needs” (children older than seven, could have some health problems and siblings).

These statistics reveal the incidence of adoption in Italy and the importance of studying it in the Italian context. 

According to Italian law, prospective adopters must be married for at least three years. There are also some limitations regarding the age differences between the adopted child and the adopters: there must be at least 18 years of age difference between the adoptive parents and the adopted child, and the maximum age difference between them is 45 years for one of the adopters and 55 for the other.

Families who desire to internationally adopt must be followed by a local accredited association that deals with all procedures regarding adoption, prepares and evaluates prospective adopters, and oversees all post-adoption procedures. One of the post-adoption procedures involves writing periodic reports on the child’s condition, which are requested by the courts of the child’s birth country and are mandatory for the families. These reports require the presence of both parents and collect several pieces of information about the child’s physical well-being and psychological condition. 

Another aspect that shows why it is important to analyze the theme of open communication about adoption-related issues in the Italian context is that, according to Italian law, the adopted child has the right to know about being adopted and to be informed about his/her past [33]. The law also details what adoptive parents should know about the child’s past, but does not specify what information parents should give to the adopted child regarding his/her past life. 

In addition, the law does not permit sharing certain information about the birth family or contact between the adoptee and the birth family until the adoptee has reached the age of 18 [33]. Nevertheless, Italian families often search for more information about adoptees’ past lives before that age [34], and Italian families seek professional support because they struggle to communicate openly [11,19]. 

The lack of information about the adopted child’s past life could be especially present in international adoption, where there may be a lack of documents sent from the child’s birth country [35,36]. 

For all these reasons, it is essential to analyze how Italian families deal with the difficulties of communicating about adoption-related themes. 

### 1.2. Aim of the Study

The current study was guided by three aims. 

Firstly, we wanted to determine whether adoptive families communicate about adoption-related themes from the beginning of their lives together as a family. For this purpose, we considered the first year after the adoption to assess whether the adopted children asked questions and shared memories about their past before being adopted. Indeed, during the first year, adopted children create an emotional bond with their adoptive parents, become more confident, and start to question their past lives. 

Secondly, we aimed to understand whether CO changes throughout the first year of adoption by exploring whether there is a difference between the first six and twelve months after placement. We chose these two time points following the laws of the children’s countries of origin.

Thirdly, we analyzed adoptive parents’ role in communication itself and the impact that adoptive parents might have on children’s willingness to communicate about adoption.

Specifically, we hypothesized that:

(a) A difference in the communication process will be present between the time points of six and twelve months in the first year of adoption. Adopted children will speak more about their past and their biological family after 12 months.
(b) Adoptive parents’ willingness to talk about their children’s adoption, history and biological families will positively affect the children’s inclination to speak about these themes. Children with adoptive parents willing to talk about adoption-related themes will be more likely to speak about such topics than children with parents who do not share information or initiate communication about these themes.

## 2. Materials and Methods

### 2.1. Participants

Our sample was composed of 537 internationally adopted children, 313 (58.3%) males, and 224 (41.7%) females. Given the nature of the study, we collected data from 1074 adoptive parents (537 mothers and 537 fathers). The children were adopted from several countries, with a mean age of 4.9 years (SD = 2.7). 

At the time of the first follow-up, the children in our sample were 5.5 years old on average. 

From the first follow-up, almost all children were healthy and presented regular physical development (*N* = 501, 93.3%) and no neurological damage (*N* = 534, 99.4%). Moreover, they demonstrated a positive relationship with their adoptive parents both at six months (*N* = 530; 98.7%) and twelve months (*N* = 531; 98.9%) after placement.

Table 1 reports sociodemographic information about the children and their parents.

### 2.2. Procedure

For children adopted in Italy from another country, the law requires a regular, periodic professional report about the child’s development that aims to track the child’s health and psychological wellbeing. Specifically, every birth country requires that Italian institutions periodically provide information about several aspects connected to adoption outcomes (e.g., physical and cognitive development, entry into the new family, school performance, awareness of one’s adoption) by sending a detailed report called a *follow-up* to the child’s birth country.

According to the Commission for Intercountry Adoption, the report can be compiled only by authorized institutions as a part of post-adoption services, and questions must be administered only to adoptive parents. All the information required by the birth country must be collected by a professional psychologist through an interview with both adoptive parents. The report cannot be compiled directly by the adoptive parents to avoid possible omissions or errors, and the adoptive parents are obliged to participate in meetings with professional psychologists [33]. Follow-up reports are mandatory for at least three years, with some countries demanding more time.

During the meeting with the adoptive family, the psychologist explores several areas connected to the adopted child’s and the family’s well-being, including how they deal with CO.

Starting from the write-up, we created an ad hoc grid, in collaboration with expert psychologists, and categorized all answers given by the adoptive parents into the coding grid. All interviews were held at two points: six months after placement (T1) and one year after placement (T2). To collect data, we followed the laws of birth countries that ask for a follow-up every six months after adoption. 

We conducted our research from 2009 to the beginning of 2018; it involved all the families who, during the period, had been helped by the Italian Center for Childhood Aid (CIAI) during the adoption process and were interviewed at the time of the first two reports, six and twelve months after they had adopted their children.

The families are comparable since the data collection procedure has been consistent across all collection steps.

We performed our study in collaboration with CIAI, an association founded in 1968 to unite families with children who are available for adoption in different countries. With more than 50 years of activity, the association aims to ensure that every adopted child receives attention, support and care, in order to help the child move towards the future. CIAI takes care of the entire adoption process, supporting families before, during and after the adoption, being one of the most important authorized institutions which can write follow-ups.

### 2.3. Measures

The coding grid we created for this study covers several areas including medical information, relationships with the adoptive family and within social contexts, communication about adoption, and the use of post-adoption services.

All areas investigated aim to understand the outcomes of adoption by exploring all its aspects, but in the current study, we focused only on communication about adoption.

In particular, this section was meant to help us to understand the levels of communication about the child’s past and adoption within the adoptive family by examining the child’s memories about his/her past life before adoption, the child’s questions about his/her past and the adoption itself, and the presence of a broader dialog about the child’s past and his/her biological family. The variables that formed this part of the questionnaire were: “child’s memories about the past”, “child’s questions about the past”, “child speaks about the biological family”, “parent speaks about the past”, and “parent speaks about the biological family”. Every variable refers to different aspects of communication:

○ *“Child’s memories about the past”* refers to the child’s memories of his/her past life before being adopted (e.g., *life with birth parents, relationships with birth relatives, life in institutions, birth culture, and language*)
○ *“Child’s questions about the past”* relates to all the child’s questions about his/her past life and adoption (e.g., *“How was I when I lived with other children?”*, *“Why did you adopt me?”)*
○ *“Parent speaks about the past”* is about the parent’s willingness to start a dialog and share information with the child about all aspects of the child’s past life except his/her relationship with birth family (e.g., *parent describes the child’s birth country or the moments before meeting the child, but without considering the birth family or the relationship between the child and his/her biological parents*).
○ *“Parent speaks about the biological family”* and *“child speaks about the biological family”* indicate discourses focused only on the biological family made by the adoptive parents and adopted child, respectively.

The Cronbach’s alpha value for this section was 0.815, showing a high level of internal consistency.

We evaluated every section of the report at six months (T1) and twelve months after the adoption (T2). 

### 2.4. Analysis Plan

We conducted statistical analysis using the IBM SPSS program, version 27 (IMB Corp, Armonk, NY, USA). To understand whether there were differences between the administrations, we performed a series of McNemar tests with all variables that formed the “communication about adoptions” part of the questionnaire (memories about the past, questions about the past, speaking about the past and speaking about the biological family).

We then performed two multivariate logistic regressions with “child speaks about the biological family” at T2 and “child’s questions about the past” at T2 as dependent variables. 

## 3. Results

### 3.1. Descriptive Analysis 

In our sample, nearly half of the children expressed memories of their past both in the first (*N* = 287; 53.4%) and in the second (*N* = 300; 55.9%) follow-up periods.

Considering questions about the past, the percentages of children who did ask about the past and children who did not were nearly equivalent at both T1 (15.3%, *N* = 82; 84.7%, *N* = 455 respectively) and T2 (17.9%, *N* = 96; 82.1%, *N* = 441 respectively).

Instead, most of the children seemed to not speak about their biological families in the first year after adoption. Indeed, six months after placement, only 100 children (18.6%) spoke about their biological families, while at twelve months, 123 (22.9%) children talked about them. 

The adoptive parents tended to communicate more about the child’s past than about the birth family, as shown in Table 2.

### 3.2. McNemar Tests

We decided to examine whether there was a significant difference in all communicative dimensions between six and twelve months by performing a series of McNemar tests.

Regarding children’s memories (McNemar test = 0.966; *p* = 0.326) and questions about the past (McNemar test = 1.625; *p* = 0.202), we did not observe any significant difference between the two administrations. Instead, we found a significant difference (McNemar test = 4.889; *p* = 0.027) in comparing the children’s willingness to communicate about their birth families at the two time points. In particular, 61 children who did not speak about their birth families at six months after adoption started to speak about them at T2, twelve months after adoption. However, there were a high number (*N* = 376) of children who did not communicate about their biological families at T1 or T2, as shown in Table 3.

Regarding the adoptive parents, we did not find a significant difference between their willingness to talk about the past (McNemar test = 0.366; *p* = 0.545) and about the birth family (McNemar test = 0.570; *p* = 0.450) during the two time points. 

### 3.3. Multivariate Logistic Regressions

#### 3.3.1. Child’s Questions about the Past

We conducted a logistic regression using ‘child’s questions about the past’ (T2) as a dependent variable, and gender, age at T2, the child’s memories at T1, and the presence at T1 of at least one adoptive parent willing to speak about the past and the biological family as independent variables. 

One year after placement, the child’s questions about the past were affected by the presence of memories as well as the parent’s willingness to communicate about the past and the birth family six months after adoption (see Table 4).

The model was statistically significant (χ^2^ (5) = 50.72, *p* < 0.001) and explained 16.4% of the variance. Children who expressed memories of their past at T1 were more likely to ask about the past one year after adoption than children who had no memories (exp (B) = 1.939; *p* = 0.042). Moreover, children with at least one adoptive parent willing to communicate about the past at T1 were more likely to question the past at T2 than those who did not have an adoptive parent inclined to speak about this theme (exp (B) = 1.898; *p* = 0.023).

Finally, children with an adoptive parent who spoke about the biological family at T1 were more likely to ask about the past at T2 than those whose adoptive parents did not communicate about the biological family at T1 (exp (B) = 2.452, *p* = 0.006).

#### 3.3.2. Child’s Speaking about the Biological Family

We conducted a logistic regression using ‘child speaks about the biological family’ (T2) as a dependent variable, and gender, age (T2), memories (T2), the presence of at least one adoptive parent willing to speak about the past (T2), and the biological family (T2) as independent variables.

The child’s communication about their biological family at T2 was affected by the child’s age, memories about the past, and the parent’s willingness to communicate about the birth family (see Table 5) at T1.

The model was statistically significant (χ^2^ (5) = 139.061, *p* < 0.001) and explained 38.2% of the variance.

Children with at least one adoptive parent willing to communicate about the biological family at six months after adoption were more likely to speak about the biological family in comparison to those who did not have an adoptive parent inclined to speak about this theme (exp (B) = 2.373; *p* = 0.017).

Moreover, children who expressed memories of their past at six months after adoption (T1) were more likely to communicate about the birth family one year after being adopted (T2) than children who did not express any memories of the past (T1) (exp (B) = 2.232; *p* = 0.015). Finally, children who were older were more likely to initiate a dialog about their birth families in comparison to younger children (exp (B) = 1.487; *p* = 0.000).

Interestingly, the presence of an adoptive parent inclined to communicate about the past did not have an impact on whether the child spoke about the birth parents (exp (B) = 0.930; *p* = 0.849), meaning that only a dialog focused on the biological family impacted the child’s willingness to speak about it.

## 4. Discussion

We examined CO during the first year of adoption, focusing on different aspects that could play a role in facilitating the communication process.

Our first aim was to understand whether adopted children ask questions, share memories, and speak about their birth families. We also wanted to comprehend whether the adoptive parents communicate about their children’s past and birth families.

Considering the adopted child, we discovered a difference between the presence of memories, questions about the past, and talking about one’s birth family. Indeed, although nearly half of the children remembered their past, only a few of them asked questions about it or talked about their birth family within the new family.

The low prevalence of dialog regarding the birth family was present not only in children but also in their adoptive parents. Almost half of the sampled children’s parents have communicated about the child’s past since the beginning, while the percentage consistently declines if we consider whether the parents speak about the children’s birth families.

These results are in line with the literature which highlights how talking about the birth family could be a challenge for adoptive families [9,20,35], especially in families who adopt from another country and often do not have any information about the birth parents. The challenge of openly communicating about adoption-related issues is often reported in the literature, based on the theory of the cycle of closed communication [10]. According to this theory, adoptive parents do not talk about adoption because they wait for their children to ask questions, but the children do not ask because they do not perceive their parents as being ready enough to communicate openly. This dynamic could obstruct CO by inhibiting questions or dialog about adoption-related issues or by reducing the frequency of open communication. This could lead family members to experience emotional distance within the family, revealing the complexity of the phenomenon and the essential role that parents play in open communication. Indeed, the cycle of closed communication could be broken if the adoptive parent is able to start a dialog and to help the child ask about their past or to share memories of it.

The small percentage of parents and children who talked about the birth family in the first year of adoption could also be explained by considering the life cycle of the adoptive family. After adoption, adoptive families are engaged in building their image as a family and all the bonds within the family itself [17], so starting open communication about the child’s past might intimidate adoptive parents [32].

Dialog about the birth family is often more frequent when the adopted child becomes an adolescent and starts to seek information and contact with his/her birth family [18,36]. Nevertheless, thoughts and questions about the birth family could start when the adoptee is a child [18,23]. Moreover, the adoptee’s ability to understand the implications of being adopted changes over time. If clear, open communication about the child’s past (also including the birth family) is present before the child’s adolescence and is appropriate to the child’s age and development, the child will experience better psychological development, fewer behavioral problems and a higher level of wellbeing within the new family [12,13,14,15]. In contrast, adoptees who grow up in families where information is hidden or there is no open communication about the child’s past are more likely to experience conflicts within the family and to feel anxiety, as well as a sense of uncertainty [28]. In the current study, the child’s age significantly impacted whether the child spoke about their birth family, and older children tended to speak about the birth family more than younger children. Moreover, our sample was composed of children of different ages, but in most cases in the period of life when, if properly guided by their parents, adopted children start to build a language of adoption and to understand of the meanings of terms related to adoption itself.

These aspects confirm that it is important to openly communicate about adoption-related issues from the beginning, especially considering that open communication is a dynamic process that follows the development of the child and the bonds created within the family [1,31]. The continued change in the communicative process needs to be taken into account when openness in adoption is studied and in clinical work with adoptive families. For this reason, we were interested in understanding whether communication would change during the first year of placement. The only aspect that significantly changed between six and twelve months after adoption was the child’s willingness to talk about his/her biological family. This variation becomes important if we also consider the factors that affect whether the child speaks about their biological family.

Children tended to communicate more about the biological family one year after the adoption if their adoptive parents had talked about the biological family six months before. Surprisingly, this effect was not present in the parent’s willingness to speak about the child’s past, meaning that only direct communication about the birth family allowed the child to express his/her thoughts about his/her birth family. The presence of one parent who communicates about a challenging theme such as the birth family seemed to be enough to increase the child’s inclination to communicate about it. This result is in line with the literature, which underlines the role of adoptive parents in guiding open communication and in helping the child understand his/her roots [4,29].

The essential role of parents in CO was also revealed by another outcome. The children tended to ask more about their past lives one year after being adopted when their parents had talked about the past and their biological families since the beginning. Living in an adoptive family where at least one parent discloses adoption-related themes encourages the child to ask questions about his/her past, thus breaking the cycle of closed communication.

All of these aspects show how important the family environment is for the adoptive child’s well-being. If the child feels and experiences a positive, warm, caring, and nurturing environment where communication is possible and the painful aspects of adoption are considered and elaborated on, he/she will be more inclined to ask, talk about and create new meanings regarding his/her history [5,9,11,14]. 

Most of the children presented regular physical development; this means that our sample was composed of healthy children with no physical impairments that could limit their ability to speak, understand and ask about adoption-related themes. Moreover, almost all of the children who formed our sample demonstrated a positive relationship with their adoptive parents from the first six months after the adoption, indicating that they were living in a family environment perceived as highly positive.

To the best of our knowledge, the current study is one of the few to explore CO during the first year of placement. In addition, we were able to study the communication of adoption-related themes with a large sample of internationally adopted children using two assessments. 

Our results could be useful both for researchers and clinicians who work with adoptive families because of the relationship we found between the adoptive parents’ willingness to talk about the birth family and the children’s inclination to do so. Given the parents’ essential role in the communication process, adoption professionals and clinicians should prepare adoptive parents to communicate from the beginning.

However, this study has some limitations. Firstly, we did not use a standardized instrument to explore the presence of open communication within the family system. This aspect could limit the generalization of the results and should be taken into account in interpreting them.

Secondly, we did not speak with the adopted children directly, but instead administered our instrument to their parents. This means that the information and aspects reported by the parents could have been mediated by them and could have been perceived differently by the children. Moreover, the children’s perceptions and willingness to speak about their past could be affected by several factors, such as their life experiences before being adopted, and could impact how they communicate within their new adoptive family. This facet should be considered when interpreting our results.

Thirdly, we focused only on two specific times of the first year from placement, so we did not explore the process of communication in each month of the first year.

Future research should use our instrument in combination with standardized instruments to increase understanding of the CO process in the first year of adoption. 

Moreover, it would be interesting to study the changes in communication by examining what happens during the twelve months after placement. This would allow researchers to have a complete overview and assessment of the discussion of adoption-related issues among the family’s members during the first year after the adoption.

Finally, future studies should examine ways to communicate about the child’s past and about his/her biological family among adoptive family members, exploring the strategies and modalities that adoptive families might use to communicate openly about these important but emotionally demanding themes. 

## 5. Conclusions

We examined CO within adoptive families during the first year of their lives together as a family. In particular, we studied CO at two different times: six and twelve months after placement to understand the child’s development and changes over time. Moreover, we analyzed adoptive parents’ role in facilitating CO within the family system. 

To the best of our knowledge, this is one of the first studies to scrutinize this theme within an Italian context, using a specific instrument that has already been employed by professional psychologists who work in the field of adoption. We collected data using follow-up information, specifically a coding grid to understand adoption outcomes and the adopted child’s wellbeing.

The follow-up is a useful, valid instrument that studies several essential aspects strictly linked to adoption, involves both adoptive parents, and is requested by law, so it facilities the collection of a large amount of data and enables clear comprehension of adoption-related aspects. Starting from this instrument, we decided to focus on elements connected with CO.

We found that, during the first year of adoption, there was a change regarding adopted children’s willingness to communicate about their birth families, while there was no difference regarding how much children questioned their past or expressed memories of it. Nevertheless, few children spoke about their birth families, which indicates that starting a dialog on this theme may be difficult for adopted children.

In investigating the role of adoptive parents, we observed that the children tended to communicate more about their biological families one year after adoption if their adoptive parents had talked about their biological families six months before. This means that adoptive parents could help their children to initiate communication about their birth families.

The knowledge developed and the concepts presented in this study can help both researchers and clinical psychologists in their work with adoptive families. These results highlight the significant relationship between adoptive parents’ willingness to talk about the birth family and children’s inclination to do so. Future research could focus on this relationship and study aspects of it in greater detail, while clinical workers could use these findings to prepare adoptive parents to communicate about adoption-related issues with their children from the beginning. 

## Figures and Tables

**Table 1:** Sociodemographic information (*N* = 537).

Children’s Socio-Demographic Information	
Gender	
Male	313 (58.3%)
Female	224 (41.7%)
Age when adopted	
Mean	4.9
SD	2.7
0–2 years	141 (26.3%)
3–5 years	172 (32%)
6–10 years	205 (38.2%)
11–13 years	12 (2.2%)
Age at follow-up	
T1	5.5
T2	6.1
Birth Country	
Burkina Faso	50 (9.3%)
Cambodia	9 (1.7%)
China	69 (12.8%)
Colombia	246 (45.8%)
Ethiopia	68 (12.7%)
India	48 (8.9%)
Thailand	23 (4.3%)
Vietnam	24 (4.5%)
Currently living	
North of Italy	309 (57.5%)
Center of Italy	68 (12.7%)
South of Italy	160 (29.8%)
Presence of siblings	
	
No siblings	163 (30.4%)
One sibling	205 (38.2%)
Two siblings	131 (24.4%)
Three siblings	34 (6.3%)
**Parents’ Socio-Demographic Information**	
Mother’s age *	
50–60 years old	196 (36.4%)
40–49 years old	266 (49.5%)
Under 40	10 (1.9%)
Mother’s level of education	
Middle school	34 (6.3%)
High School	177 (33%)
College	326 (60.7%)
Father’s age **	
50–60 years old	128 (23.8%)
40–49 years old	306 (57%)
Under 40	37 (6.9%)
Father’s level of education	
Middle school	63 (11.7%)
High School	240 (44.7%)
College	234 (44.7%)

* NA: 65 (12.2%). ** NA: 66 (12.3%).

**Table 2:** Adoption-related issue communication dimensions in both children and parents.

	After Six Months (T1)	After Twelve Months (T2)
Child’s memories about the past		
Yes	287 (53.4%)	300 (55.9%)
No	150 (46.6%)	237 (44.1%)
Child’s questions about the past		
Yes	82 (15.3%)	96 (17.9%)
No	455 (84.7%)	441 (82.1%)
Child speaks about the biological family		
Yes	100 (18.6%)	123 (22.9%)
No	437 (81.4%)	414 (77.1%)
Adoptive Parent speaks about the past		
Yes	228 (42.5%)	237 (44.1%)
No	309 (57.5%)	300 (55.9%)
Adoptive Parent speaks about the biological family		
Yes	73 (13.6%)	81 (15.1%)
No	464 (86.4%)	456 (84.9%)

**Table 3:** Child’s speaks about the biological family: A comparison between the two time points.

Child Speaks about Biological Family	One Year after Adoption (T2)	
Six months after adoption (T1)	Yes	No
Yes	62	38
No	61	376

N = 537.

**Table 4:** Multivariate logistic regression of child’s questions about the past (0 = “yes”; 1 = “no”).

Variable	B	Exp (B)	SE
Gender	−0.215	0.807	0.248
Age at T2	0.071	1.074	0.053
Child’s memories T1	0.662 *	1.939	0.326
Parent speaks about the past T1	0.641 *	1.898	0.282
Parent speaks about the biological family T1	0.897 *	2.452	0.329

* *p* < 0.05.

**Table 5:** Multivariate logistic regression of ‘child speaks about the biological family’ (0 = “yes”; 1 = “no”).

Variable	B	Exp (B)	SE
Gender	0.049	1.051	0.259
Age at T2	0.396 ***	1.487	0.057
Child’s memories T1	0.803 **	2.232	0.331
Parent speaks about the past T1	−0.072	0.930	0.298
Parent speaks about the biological family T1	0.864 **	2.373	0.363

** *p* < 0.05, *** *p* < 0.001.

## Data Availability

Not applicable.
